# Impact of Hydrogen Voiding in Chip-to-Chip Electroless All-Copper Interconnections

**DOI:** 10.3390/mi15050612

**Published:** 2024-04-30

**Authors:** Nana Ren, Yuyi Zhang, Wenlong Shu, Chenxiao Lu, Wenjing Zhang, Zhuo Chen, Fuliang Wang

**Affiliations:** 1 College of Mechanical and Electrical Engineering, Central South University, Changsha 410083, China; 213712087@csu.edu.cn (N.R.); 213712119@csu.edu.cn (Y.Z.); 223711050@csu.edu.cn (W.S.); 223712193@csu.edu.cn (C.L.); wangfuliang@csu.edu.cn (F.W.); 2 The School of Aerospace Engineering and Applied Mechanics, Tongji University, Shanghai 200092, China; zhangwenjing@tongji.edu.cn

**Keywords:** 3D integration, electroless copper interconnection, hydrogen-voiding, microfluidic channel device

## Abstract

Three-dimensional (3D) integration has become a leading approach in chip packaging. The interconnection density and reliability of micro-bumps in chip stacking are often threatened by high bonding temperatures. The method of building chip-to-chip interconnections by electroless deposition of metal has its distinct merit, while the interfacial defect issue, especially that related to voiding during the merging of opposite sides, remains largely unsolved. In this study, to trace the influencing factors in the voiding, the growth characteristics of the electroless all-copper interconnections were examined by carrying out deposition experiments in a microfluidic channel device. The results show that when the gap between the opposite copper bumps to be electrolessly merged is as low as 10 μm, significant voids appear at the inflow side and the top of the copper bumps because the hydrogen cannot be expelled in time. A finite-element flow model of the plating solution between the chips was established, which showed that the flow rate of the plating solution around the copper bumps was much higher than in the merging gap, causing an uneven supply of reactants. Based on these findings, we proposed two potential solutions, one is to improve the flow mode of the plating solution, and the other is to add the reaction inhibitor, 2,2′-bipyridine. Finally, the combination of these two approaches successfully achieved an improved merging quality of the copper joints.

## 1. Introduction

With the development of electronic products for miniaturization, the exploration of small-pitch packaging technology has become a research hotspot [1]. The area array-type fine-pitch chip-to-substrate interconnection, or those implemented in a three-dimensional fashion commonly known as vertical chip-stacking or 3D integration, offer elevated I/O capabilities and enhanced processing speeds. The core of this advanced packaging lies in the establishment of metal wiring. In addition to providing electrical connections between integrated circuits and PCB boards, interconnection technology also needs to take on a certain amount of mechanical support and heat dissipation functions [2]. The traditional flip-chip interconnection commonly employs spherical tin solder. However, due to the low electric migration resistance of the solder itself, this leads to significant migration of metal ions in the metal layer, forming local voids or accumulations [3]. On the other hand, during the interconnection process of solder joints, reactions involving metals such as Sn, Ag, and Cu often result in the formation of intermetallic compounds (IMC) and their poor mechanical properties, directly posing a threat to the reliability of the interconnections and failing to meet the requirements for effective 3D integration [4,5]. Additionally, most solder joint interconnection technologies are conducted under high-temperature conditions, typically around 250 °C. However, the different coefficients of thermal expansion (CTE) among various materials inside the packaged device can lead to significant thermal stress and strain at the interconnections, consequently diminishing the product’s life [6,7].

Due to its high electrical conductivity, low resistivity, and excellent reliability, copper has found widespread applications in the semiconductor industry. Copper-to-copper direct interconnection can avoid the low reliability and thermal mismatch problems associated with solder joint interconnections. Methods like insertion bonding [8,9], surface-activated bonding [10], as well as Cu-nanoparticle sintering, have been extensively developed, each meeting specific application requirements [11]. Recently, electroless plating interconnection has become a promising alternative to address the issues in current Cu–Cu bonding attempts. By depositing target metals on pre-aligned chip bumps through a chemical reaction, the resulting joints exhibit superior mechanical as well as electrical characteristics. Electroless copper interconnection was first proposed for the interconnection from chip to substrate [12] but is also viable in chip-stacking 3D integration. The strength of the copper joints can reach 100 MPa after annealing. In addition to the ultra-high strength of the copper joints, electroless deposition interconnection technology enjoys several other advantages, including superior electrical properties and the elimination of process pressure, as well as alignment tolerance. [13]. Metals other than Cu are also developed as the material for electroless interconnections, including Ni-P [14,15], NiB [16], and Au [17,18], but Cu remains a favorable choice in 3D integration [19].

In the research of chemical deposition interconnection, defects are often observed between the merging copper bumps due to incomplete copper deposition. This is because when the pre-alignment gap of copper bumps is less than 10 μm, deposited metal tends to accumulate at the edge rather than the top of the copper bumps [20]. Consequently, this leads to interconnection voids, reducing the reliability of the interconnection. In recent studies, a microfluidic channel device that enhances material transport and reaction efficiency by controlling the directional flow of the plating solution was employed, facilitating metal growth at the top of the copper bumps [21]. However, they found that voids still exist when the tops of the copper bumps are flat. This intrinsic drawback was improved when the geometric shape of the copper bumps was deliberately altered from flat-topped to dome-shaped [22]. It should be noted that the existing studies of electroless all-Cu interconnections mostly used formaldehyde as the reductant, while other deposition systems, such as the ones that use hypophosphite or glyoxylic acid, were seldom reported. Besides the merit of being environmentally friendly, hypophosphite often reduced Cu to nucleate at a much higher rate than formaldehyde, and the deposited Cu exhibited varied morphology with the addition of certain crystallization modifiers, potentially facilitating the interfacial merging. However, the side reaction of hydrogen generation is a significant issue that could jeopardize the feasibility of it being practically adopted. In this study, to investigate the voiding issues in a chip-to-chip electroless Cu interconnection using hypophosphite as a reductant, we carried out experimental works including cross-sectional observations of the interconnections formed in a microfluidic channel device and the numerical calculation of the solution flow characteristics. Influences of deposition parameters such as the gap height of the merging bump pair, the solution flow mode, as well as the addition of reaction inhibitor, were investigated. As almost no additive was added to the plating solution, this study is essentially a verification under a harsh condition for electroless interconnection, which could give rational guidance to the optimization of the process in a general sense.

## 2. Materials and Methods

**Figure 1:**
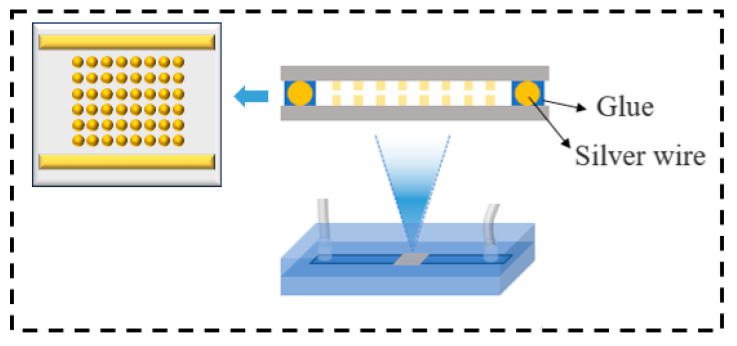
Schematic diagram of electroless copper plating experiment.

The chip size used in this study is 6 mm × 6 mm × 0.5 mm, and the chip is arranged with an array of copper bumps in a 14 × 16 pattern. The diameter of the copper bumps is 40 μm, the height is 35 μm, and the pitch is 160 μm. Figure 1 is the schematic diagram of the electroless copper plating configuration. Firstly, the chips are aligned through an Athlete CB-600 flip-chip bonder(Athlete, Nagano, Japan). Silver wires with different diameters are then used to control the interconnection gap between the two copper bumps, and adhesive is applied to temporarily cure the aligned stack. Subsequently, the pre-aligned chips are placed inside the microfluidic channel device, and a peristaltic pump is used to quantitatively deliver the plating solution to enable the deposition on the surface of the copper bumps. In order to ensure uninterrupted and effective chemical reactions, preliminary treatment of the chips is essential. The pretreatment process consists of two steps, degreasing using anhydrous ethanol, and removing surface oxides using sulfuric acid. After pretreatment, activation is carried out using a palladium chloride aqueous solution with a concentration of 0.4 g/L. The flow rate for all these steps is set at 10 mL/min, with a duration of 5 min each. The plating solution is maintained at a temperature of 70 °C, and the composition of the plating solution is shown in Table 1, all reagents are from Macklin, Shanghai, China. The chips after interconnection were ground to the cross section of the interconnection copper bumps; the cross section of the copper bumps was observed by a TESCAN Vega 3 (TESCAN, Brno, Czech Republic) scanning electron microscope.

## 3. Results and Discussion

### 3.1. Three Stages of Electroless Copper Plating

**Figure 2:**
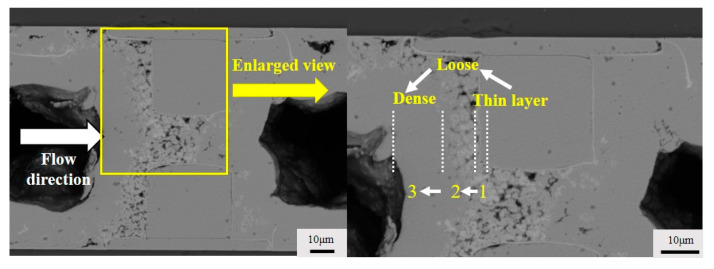
Three stages of electroless copper plating.

**Figure 3:**
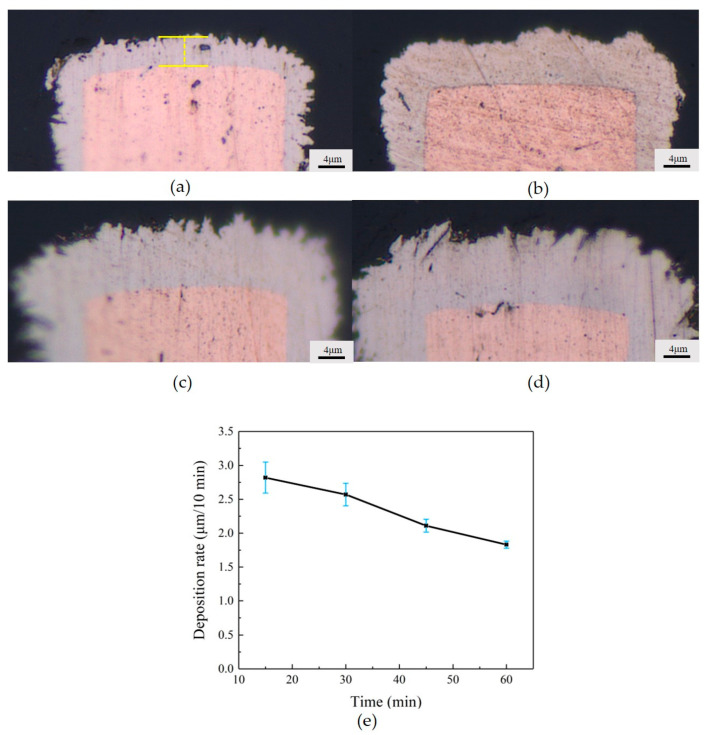
Thickness of electroless copper plating at different times: (**a**) 15 min; (**b**) 30 min; (**c**) 45 min; (**d**) 60 min; (**e**) plot of deposition rate change with time.

We observed three stages of chemical deposition of copper on the inflow side of the copper bumps, as shown in the cross-sectional images in Figure 2. In the first stage, the copper grew a thin and dense layer around the copper bump; in the second stage, the deposited copper became sparse, and voids between the copper bumps significantly increased; in the third stage, the copper continued to deposit, transitioning once again from sparse to dense. The reasons for the formation of these three stages were analyzed. The first stage occurs during the activation of the copper bumps when a uniform layer of palladium atoms attaches to the surface. Palladium possesses strong catalytic activity, leading to a relatively rapid rate of chemical deposition of copper during the initial stage [23]. To better explain the reasons for the formation of the second and third stages, we studied the average deposition rate of electroless deposition on single chips immersed in bulk deposition plating solution at 15 min, 30 min, 45 min, and 60 min of plating time, as shown in Figure 3. Voids do not occur in the single chip deposition samples since the hydrogen gas easily escapes without the constraint of the narrow gap. The deposited layer showed an uneven profile with some local thickness variation, which is a result of the large grain size since no grain refiner was added to the pristine deposition plating solution. The central position at the top of the copper bump is selected as the measurement point, as shown in Figure 3a. We calculated that the overall deposition rate of chemical copper plating decreases with the increase in reaction time, as shown in Figure 3e. This could be attributed to the continuous consumption of reactants around the copper bumps, leading to a significant decrease in local reactant concentration and, consequently, the gradual slowing down of the reaction rate. Therefore, we concluded that the significant appearance of voids in the second stage of copper deposition is primarily due to the rapid deposition rate of the copper. The flow of the plating solution struggles to expel all the generated hydrogen, leading to the residual presence of some hydrogen, hindering the further growth of copper and resulting in the formation of voids. In the third stage, the copper becomes dense again because, as the reaction continues, the deposition rate of the copper gradually decreases, and the hydrogen is mostly expelled, eliminating the presence of voids.

### 3.2. The Impact of the Interconnection Gap on the Formation of Voids

**Figure 4:**
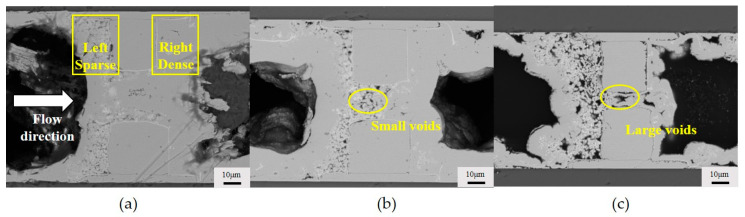
Cross section of copper bumps under different interconnection gaps: (**a**) 35 μm; (**b**) 20 μm; (**c**) 10 μm.

We studied the quality of copper joints with an interconnection gap of 10–35 μm. As can be seen from Figure 4, we observed that when the interconnection gap is 35 μm, the copper at the interconnection is dense, with almost no voids. As the interconnection gap decreases to 20 μm, some small voids appear at the tops of the copper bumps. Further reducing the interconnection gap to 10 μm results in the emergence of widespread voids between the copper bumps. This is because the flow of the plating solution was limited by the reduced interconnection gap. Consequently, the flow tends to gravitate towards the sides of the copper bumps rather than their tops.

**Figure 5:**
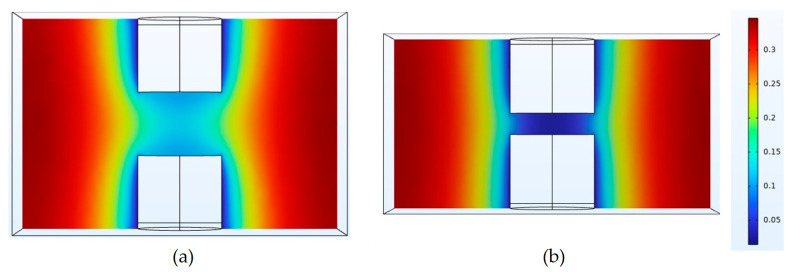
The flow rate (m/s) of plating solution with different interconnection gaps: (**a**) 30 μm; (**b**) 10 μm.

To gain a more in-depth understanding of this phenomenon, we established a numerical model of the plating solution flow during the chip interconnection process, as shown in Figure 5. The model simulated the flow state and velocity of the plating solution under different interconnection gaps. The inlet and outlet boundary conditions were set the same as in the numerical model. This observation indicates a notably higher flow rate of the plating solution on both sides of the copper bumps compared to the top. At the same time, it can be observed that the flow rate of the plating solution at the top of the copper bumps also decreases with the decrease in the interconnection gap.

Theoretically, the constrained plating solution flow has dual impacts on the deposition efficacy. Firstly, it hinders the timely provision of reactants required for the process. Therefore, the rate of supply of active ions around the copper bumps increases, resulting in a higher thickness of deposited copper over the same period, further exacerbating the formation of voids at the tops of the copper bumps. Secondly, it hampers the efficient expulsion of hydrogen gas generated during the electroless copper interconnection experiment. During the electroless copper interconnection experiment, a substantial amount of hydrogen is generated on the surface of the copper bumps. The expulsion of this hydrogen gas relies solely on the flow of the plating solution. If the timely discharge of hydrogen is impeded, it also will limit the growth of the copper.

We also observe that, under all the interconnection gap conditions, compared to the dense deposition of copper on the right side, there are numerous voids on the left side of the copper bumps. This is because we set the direction of the plating solution flow to be from left to right. The by-product hydrogen generated on the left side of the copper pillar is hindered by the copper bumps during the discharge process, leading to accumulation and the formation of voids. In contrast, the hydrogen on the right side can be smoothly expelled, relying on the flow of the plating solution.

Based on the above research, we conclude that the voids in the copper joints are generated due to the presence of hydrogen. To reduce or eliminate these voids, two methods can be employed: (1) by controlling the flow of the plating solution to ensure uniform flow between chip–chip or chip–substrate interfaces; (2) by utilizing the inhibitory effect of a certain additive to decelerate the generation of hydrogen.

### 3.3. Optimization of the Flow Mode of the Plating Solution 

**Figure 6:**
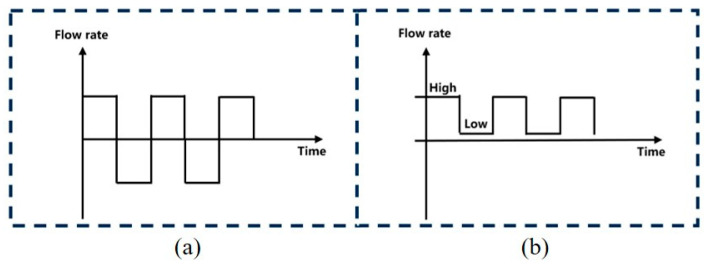
The flow mode of plating solution: (**a**) oscillating flow; (**b**) intermittent flow.

**Figure 7:**
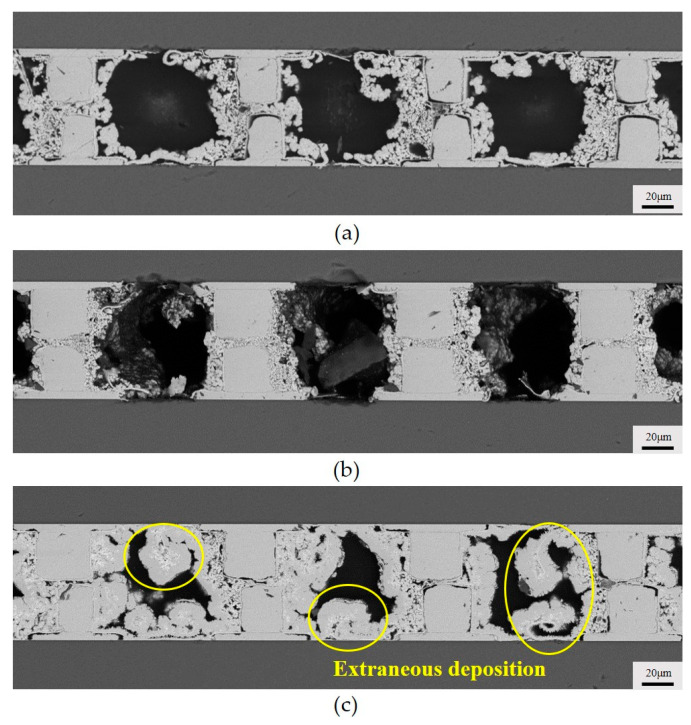
Cross section of copper bumps at different oscillating flow: (**a**) 30 s; (**b**) 1 min; (**c**) 10 min.

To diminish the voids in the copper joints, the plating solution’s flow mode has been optimized. Figure 6 shows schematic diagrams of two flow patterns: oscillating flow and intermittent flow. In both flow modes, we achieved an abrupt change in flow velocity or direction to apply a sudden force to the generated hydrogen, increasing the probability of the hydrogen being expelled. We evaluated the quality of the copper joints under different flow modes by altering the oscillating or intermittent time. Figure 7 illustrates the cross sections of copper joints under different oscillating intervals. It can be observed that when the time interval is 30 s, the deposited copper on both sides of the copper bumps is very sparse. With an oscillation interval of 1 min, there are almost no large voids in the copper joints. This indicates that oscillating flow can increase the probability of the plating solution flowing over the tops of the copper bumps. On the one hand, it enhances the timely supply of reactants, and on the other hand, it helps to expel the hydrogen gas at the tops of the copper bumps. However, excessively frequent changes in flow direction may lead to an increase in voids in the deposited copper. 

It is worth noting that under most conditions, the deposition not only took place on the pre-arranged Cu micro-bumps but also on the blank Si surface, which is unwanted, namely the extraneous deposition. One exception is that, compared to the continuous flow of copper deposition, there is almost no extraneous copper deposition on the Si substrate under the oscillating flow. This is owing to the significant suppression of the overall reaction. When the oscillation interval is 10 min, there are still numerous voids between the copper bumps and noticeable one-sided sparse voids due to the direction of plating solution flow, and evident extraneous deposition on the Si similar to that under continuous flow could be observed.

There are several possible causes for extraneous deposition on the Si, and we can roughly summarize them into two categories: (1) palladium catalyst from the activation solution adsorbed on the Si surface; (2) the spontaneous decomposition of the plating solution generated Cu particles that could be deposited to the unwanted area. Some researchers have suggested this to be connected to hydrogen generation, either in the form of hydrogen gas or hydrogen atoms; either could replace the cupric ion in the solution [14].

**Figure 8:**
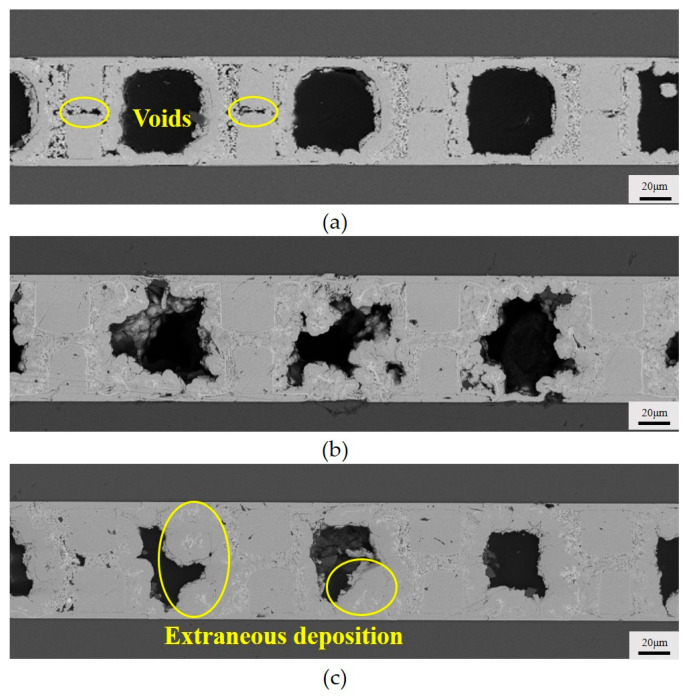
Cross section of copper bumps at different intermittent flows: (**a**) 3 s; (**b**) 5 s; (**c**) 7 s.

For the intermittent flow, we set the high flow velocity of the plating solution to be 15 mL/min and the low flow velocity to be 0.2 mL/min. It can be observed in Figure 8 that as the intermittent time increases from 3 s to 7 s, the voids in the copper joints gradually decrease, even disappear, and the deposited copper becomes denser. This may be attributed to the increased intermittent time, in which the high flow velocity of the plating solution can enhance the expulsion of hydrogen gas. At the same time, with the increase in the intermittent time, more copper is deposited on the Si.

In conclusion, we found that the change in flow mode alone alleviated, to some extent, the formation of voids but did not effectively change the extraneous deposition on the Si. To achieve copper joints without extraneous deposition on Si and voids, further optimization of the plating solution composition is required. This can be achieved by leveraging the inhibitory effect of 2,2′-bipyridine itself to decelerate the generation of hydrogen gas.

### 3.4. Optimization of the Composition of the Plating Solution 

**Figure 9:**
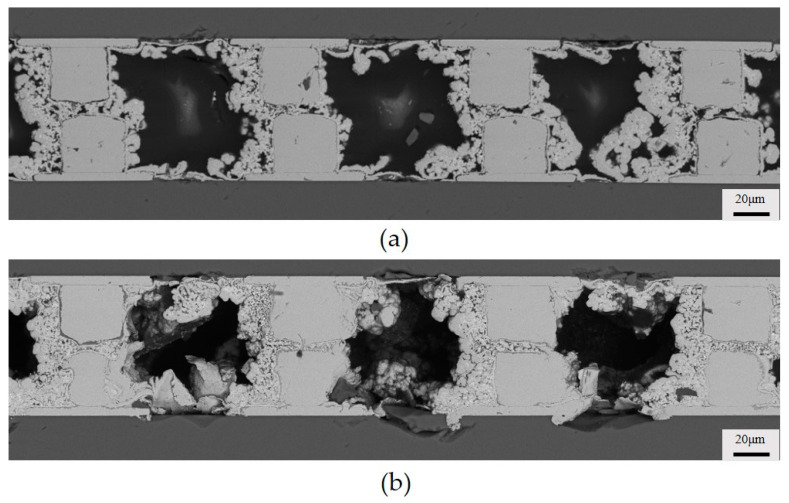
Cross section of copper bumps at different concentrations of 2,2′-bipyridine in continuous flows: (**a**) 5 mg/L, 10 mL/min; (**b**) 10 mg/L, 5 mL/min.

Through the above study, we learned that improving the plating solution flow mode can only mitigate the formation of voids to some extent. However, dense copper deposition always occurs simultaneously with extraneous deposition. Based on this, we can reduce or even eliminate voids by adding inhibitors to the plating solution to lower the chemical copper deposition rate while improving the uniformity of the chemical copper deposition. 2,2′-bipyridine, as a common chelating agent, can make the copper deposition dense and bright while also reducing the nickel content [24]. The cross-sectional views of the copper joints under different 2,2′-bipyridine concentrations are shown in Figure 9. When the additive concentration is 5 mg/L, and the flow velocity is 10 mL/min, the void is still evident and relatively large, and there is also some extraneous deposition on the Si. When the concentration of 2,2′-bipyridine is 10 mg/L, and the plating solution is set to a continuous flow of 10 mL/min, the electroless copper interconnection fails, resulting in little deposited copper on the chip. This failure occurs because 2,2′-bipyridine acts as an inhibitor, and increasing its concentration inhibits the reduction of copper. Additionally, the excessive flow rate of the plating solution disturbs the stability of the chemical reaction. Therefore, it is necessary to reduce the flow rate of the plating solution. As the additive concentration continues to increase to 10 mg/L, with a flow rate of 5 mL/min, the voids on the left and top of the copper bumps are relatively reduced, the deposited copper on the right side of the copper bumps is relatively dense, and the extraneous deposition on the Si is also significantly reduced. The inhibitor not only suppresses the growth rate of deposited copper but also inhibits the growth of copper on the Si.

In addition, we observed that when the additive concentration was 15 mg/L, and the flow velocity was set to 2 mL/min, the interconnection always failed, and there was no copper deposition on the copper bumps. This may be due to the excessive flow rate of the plating solution, which disturbs the reaction of electroless copper plating.

**Figure 10:**
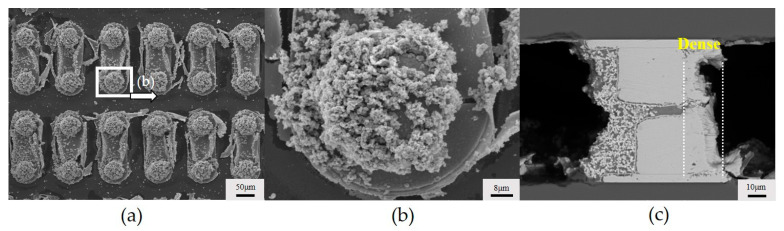
Cross section of copper bumps with a concentration of 15 mg/L of 2,2′-bipyridine in a continuous flow: (**a**) failed; (**b**) enlarged view; (**c**) successful.

However, when the flow velocity was set to 0.2 mL/min, the interconnection of some sample chips failed, but the copper bumps were not completely free of deposited copper, as shown in Figure 10a,b that the deposited copper is in the form of fine particles. The cross section of the successfully interconnected sample copper bumps is shown in Figure 10c. There are fine copper particles on the left side of the copper bump and a dense layer of deposited copper on the right side of the copper bump. This shows that when the flow rate of the plating solution is too slow, the hydrogen on the left side is more difficult to discharge than that on the right side of the copper bumps, and the growth of deposited copper is hindered.

Based on the above conclusions, we deduce that the addition of 2,2′-bipyridine stabilizes the plating solution, necessitating a slower flow velocity to sustain continuous reactions. Meanwhile, it is crucial to ensure that the continuously generated hydrogen gas does not linger between the copper bumps for an extended period. Consequently, we optimized the flow mode of the plating solution based on an optimized plating solution.

**Figure 11:**
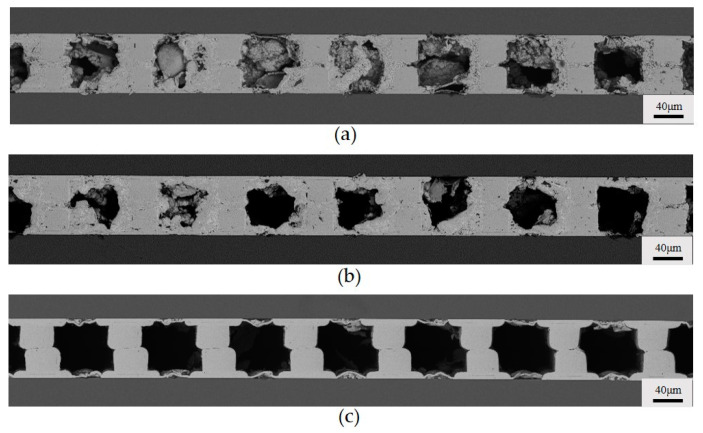
Cross section of copper bumps with different concentrations of 2,2′-bipyridine in an intermittent flow, (**a**) 5 mg/L; (**b**) 10 mg/L; (**c**) 15 mg/L.

When the concentration of 2,2′-bipyridine in the plating solution is 5 mg/L and the flow mode is an intermittent flow with an intermittent time of 5 s, the deposited copper at the interconnection is relatively concentrated on the left side; that is, the inflow direction of the plating solution and the deposited copper on the right side of the copper bumps are inhibited, as shown in Figure 11a.

When the concentration of 2,2′-bipyridine was increased to 10 mg/L, it was difficult to obtain a successful interconnection sample at a temperature of 70 °C. This may be due to the fact that the plating solution requires a relatively stable deposition environment after the additives are added, and the plating solution fluctuates greatly in the intermittent flow mode, which makes the chemical reaction more difficult. Therefore, the plating solution temperature was raised to 75 °C. As shown in Figure 11b, the deposited copper at the copper joints becomes dense, but, due to the increase in temperature, it also leads to more extraneous deposition on the Si.

The plating solution temperature and flow mode remained unchanged, and the 2,2′-bipyridine concentration continued to increase to 15 mg/L. The deposited copper was very dense, and the interface between the deposited copper and the copper bumps almost disappeared, as shown in Figure 11c. The extraneous deposition on the Si can still be observed but is much alleviated. This is the case where the first cause of the extraneous growth appears: the physisorption of a trace amount of Pd catalyst particles on the Si surface. Therefore, proper surface passivation treatment is needed for the complete elimination of such unwanted Cu growth. In summary, when the concentration of 2,2′-bipyridine was increased to 15 mg/L, the electroless copper plating interconnection experiment showed the highest interconnection quality.

It is well known that the electroless deposition characteristics of Cu can be altered by the addition of certain kinds of additives. Besides the 2,2′-bipyridine investigated herein, the effects of other organic compounds such as thiourea or potassium ferrocyanide were also reported previously. It is believed that the oxidation of hypophosphite could be somewhat tempered by such substances, which slow the hydrogen-generating side reaction. In this sense, the deposition reaction rate is at least partially controlled by the supply of hypophosphite [25]. Further controlling of the deposition uniformity, therefore, could start with the manipulation of the sub-steps in the overall deposition reaction, such as the cathodic reduction of Cu^2+^, the anodic oxidation of hypophosphite, the catalytic activity of the Ni element, or even the crystallization of the reduced Cu. The possible participation of a zero-valence H atom or H_2_ in the reduction of Cu^2+^ into Cu^0^ should also be considered.

## 4. Conclusions

This electroless copper interconnection can achieve high-density copper bump interconnections at low temperatures. The growth stage of electroless copper plating on copper bumps and the cross-section morphology of copper joints were studied by using a microfluidic channel device.

1. On the inflow side of the copper bumps, the chemical deposition of copper undergoes three stages, which are closely related to the activation of the copper bumps and the deposition rate of copper.
2. When the interconnection gap decreases to 10 μm, extensive voids appear around the copper bumps. The formation of these voids is related to the direction of the plating solution flow, with voids being more prone to occur on the inflow side of the copper bumps rather than the outflow side.
3. Improving the plating solution flow mode can only partially alleviate the generation of voids, and the root cause of void formation lies in the hydrogen gas that cannot be expelled in time. Extraneous deposition on the Si is only suppressed when the deposition is significantly suppressed.
4. The addition of 2,2′-bipyridine can decelerate the generation of hydrogen gas, and when combined with the optimized plating solution flow mode, the quality of the interconnection can be improved.

## Figures and Tables

**Table 1:** Composition of the plating solution.

Composition of the Plating Solution	Concentration (g/L)
CuSO_4_ 5H_2_O	10
NaH_2_PO_2_∙H_2_O	30
H_3_BO_3_	30
NiSO_4_ 6H_2_O	2
Na_3_C_6_H_5_O_7_ 2H_2_O	23.5
PEG	1.28
2,2′-bipyridine	0.005, 0.01, 0.015

## Data Availability

The original contributions presented in the study are included in the article, further inquiries can be directed to the corresponding author.
